# Relationship between *SCN5A* gene H558R polymorphism and atrial fibrillation in Tibetan and Han nationalities at high altitude

**DOI:** 10.1097/MD.0000000000025229

**Published:** 2021-03-26

**Authors:** Jiang Liu, Fengcai Yao, Kaiyue Han, Jinping Chai, Dekuan Tian, Jinwei Zhang, Rong Wang, Wei Li, Yanmei Shen, Yuanfeng Ma, Sang Geng, Xiaoling Su

**Affiliations:** a Department of Cardiology, Xi ’an International Medical Center Hospital, Xi ’an, Shaanxi; b Department of Cardiology, Qinghai Provincial People's Hospital; c Department of Cardiology, Shanghai Fifth People's Hospital, Shanghai; d Graduate School of Qinghai University; e Qinghai University; f Department of Cardiology, Qinghai Red Cross Hospital; g Qinghai Cardiovascular and Cerebrovascular Disease Hospital; h People's Hospital of Hainan Tibetan Autonomous Prefecture, Xining, Qinghai, China.

**Keywords:** atrial fibrillation, H558R, high altitude, *SCN5A* gene

## Abstract

This study aimed to explore the relationship between H558R polymorphism of the *SCN5A* gene and atrial fibrillation (AF) in Tibetan and Han nationalities at high altitude.

A total of 50 Tibetan and 50 Han patients with AF at the same altitude (2260 m) were included. Meanwhile, the general clinical data of patients without AF (50 Tibetan and 50 Han) matched with the data of patients with AF were included during the same period. The blood samples of patients were collected to extract DNA. The DNA sequencing was performed by Xi’an Zhenpin Biotechnology Co., Ltd. The mutation loci of the sequence were located and identified by DNA sequencing. The general information, laboratory examination, color Doppler echocardiography, and genotypes and alleles of each group were analyzed. The multivariate logistic regression analysis was used to determine the independent risk factors for AF.

The genotype and allele frequencies of the H558R locus of the *SCN5A* gene in the AF groups of Tibetan and Han nationalities were significantly different from those in the non-AF groups (*P* < .05). The genotype and allele frequency of the H558R locus of the *SCN5A* gene in the AF group of Tibetan nationalities were not significantly different from those in the AF group of Han nationalities (*P* > .05). The logistic regression analysis of the total population revealed that coronary heart disease, age, total cholesterol (TC), left atrial diameter, and G allele were independent risk factors for AF occurrence.

The occurrence of AF in Tibetan and Han nationalities at high altitude is associated with the polymorphism of H558R locus of the *SCN5A* gene. The G allele is an independent risk factor for the occurrence of AF in Tibetan and Han nationalities.

## 1 Introduction

Atrial fibrillation (AF) is one of the most common tachyarrhythmias in clinic. It causes complications, such as heart failure, thromboembolism, stroke. At present, various treatments are available for AF, but its pathogenesis is still unclear. Recent studies have shown that gene mutations are closely associated with AF occurrence.^[1,2]^ Several ion channel protein genes have been found to be associated with AF, including sodium, potassium, and calcium ion channels. Gene mutations cause not only AF but also some other arrhythmias, such as sick sinus syndrome, long QT syndrome, early repolarization syndrome, Brugada syndrome. The occurrence of these arrhythmias is associated with enhanced or absent ion channel function.

However, mutations in cardiac sodium channels are closely related to the occurrence of AF. Cardiac sodium channels are mainly encoded by the *SCN5A* gene in human cardiomyocytes.^[3]^ Previous studies indicated that H558R polymorphism of the *SCN5A* gene was associated with lone AF.^[4]^ Similarly, mutations in the *SCN5A* gene have been reported to change the function of sodium channels, leading to a further reduction in sodium influx, shortening of action potential duration, and then arrhythmia.^[5]^

Recent studies on high-altitude areas have found that the results of the same disease in different ethnic groups are different.^[6–8]^ However, at present, no report exists on the study of patients with AF in different ethnic groups at high altitude. Therefore, based on the special geographical environment of high altitude, we aimed to investigate the relationship between H558R polymorphism of the *SCN5A* gene and AF in Tibetan and Han nationalities at high altitude and the independent risk factor for AF occurrence.

This study uses a case-control study, it collects the general baseline data of AF and non-AF of the Tibetan and Han nationalities at the same altitude and samples of the *SCN5A* gene H558R locus polymorphism. After statistical analysis of the independent risk factors for AF, nomogram is used to make a prediction model of AF.

## 2 Materials and methods

### 2.1 Participants

Patients with AF were consecutively selected from the Cardiac Interventional Department of Qinghai Provincial People's Hospital from May 2018 to May 2019, including 50 Tibetan patients and 50 Han patients. Meanwhile, the general clinical data of patients without AF (without other arrhythmias) of Tibetan and Han nationalities (50 for each) matched with the data of patients with AF during the same time period were consecutively included.

The inclusion criteria were as follows: patients with a definite diagnosis of AF detected using 24-hour dynamic electrocardiogram or 12-lead electrocardiogram; patients aged between 18 and 90 years; and patients who neither had other blood relationships nor a family history of heterogamy. The exclusion criteria were as follows: incomplete clinical data; congenital heart disease; valvular heart disease; severe liver and kidney dysfunction; malignant tumors; blood, rheumatic immunity, and other diseases; hyperthyroidism and hypothyroidism; acute and chronic inflammatory diseases; cardiomyopathy; and chronic pulmonary heart disease and chronic obstructive pulmonary disease.

### 2.2 Ethical approval

This study was approved by the Medical Ethics Committee of Qinghai Provincial People's Hospital. Before collecting blood samples, each patient agreed and signed the informed consent form.

### 2.3 Definitions and data collection

The professional medical history of participants was recorded, and the physical examination was conducted by professional cardiologists after admission. Sex, age, smoking history, drinking history, and presence/absence of associated underlying diseases (hypertension, type 2 diabetes, and coronary atherosclerotic heart disease) were also recorded. On the morning of the second day, about 3 mL of fasting venous blood was withdrawn and collected in an ethylene diamine tetraacetic acid (EDTA) anticoagulant tube and stored in a –80 °C refrigerator for DNA extraction. Meanwhile, relevant blood indices, including white blood cell (WBC) count, red blood cell (RBC) count, hemoglobin (HB) content, red blood cell distribution width (RDW-SD, RDW-CV), platelet (PLT) count, and the levels of fasting blood glucose (FBG), triglyceride (TG), total cholesterol (TC), high-density lipoprotein (HDL), low-density lipoprotein (LDL), uric acid (UA), thyroid-stimulating hormone (TSH), free triiodothyronine (FT3), free thyroxine (FT4), and C-reactive protein (CRP) were measured. Cardiac color Doppler ultrasound, including left atrial diameter (LAD), left ventricular end-diastolic volume (EDV), interventricular septal thickness (IVST), left ventricular posterior wall thickness (LVPWT), left ventricular ejection fraction (LVEF), and mean systolic and diastolic pressures, through 24-hour ambulatory blood pressure monitoring, was performed by the same doctor in the cardiac interventional department.

The DNA was extracted by professionals and purified to determine the concentration in the pathology and molecular laboratory of Qinghai Provincial People's Hospital.

The content of DNA was determined using a Boao microspectrophotometer (Beijing Boao Jingdian Biotechnology Co., Ltd. Product model: NanoQ Micro). A sample of 2 μL was added to the cell with a pipette. The absorbance was measured at 260–280 μm. The purity of DNA was 1.8–2.0, and the concentration was 10–25 μg/μL.

The primer sequence was as follows:

SCN5A-H558R-F GCCAGTGGCACAAAAGACAGGCT
SCN5A-H558R-R GGAACTGCTGATCAGTTTGGGAGA

The DNA sequencing was performed by Xi’an Zhenpin Biotechnology (No.298 Keji Road, Yanta District, Xi’an) Co., Ltd. The mutation loci of the sequence were located and identified by DNA sequencing.

Genotyping of *SCN5A*-H558R polymorphism:

After DNA extraction (Beijing Yaanda Biotechnology Co., Ltd., catalog number: DP348), primer design, sequencing, endonuclease selection and electrophoresis, the genotypes of H558R locus of SCN5A gene were determined to be AA, AG, GG.

### 2.4 Statistical analysis

Statistical analysis was performed using SPSS24.0 software (SPSS 24.0 for Windows, SPSS Inc., IL) and R 3.4.3 (The R Foundation, Vienna, Austria). The normality test (Shapiro-Wilk) was carried out for the original variables, and the independent-samples *t* test was selected for comparing data with a normal distribution. Two independent samples were selected for nonnormal distribution to compare the differences using the nonparametric test. Chi-square test is used for count data and Hardy–Weinberg balance test; multivariate logistic regression was use to analyze independent risk factors for AF; *P* < .05 is statistically significant; the nomogram is then used to make a prediction model for the occurrence of AF.

## 3 Results

Significant differences were found in age, drinking history, associated coronary heart disease, RDW_SD, RDW_CV, PLT, TG, TC, HDL, LDL, FT3, CRP, LAD, IVST, and LVPWT between the AF and non-AF groups of Tibetan patients (*P* < .05). Also, significant differences were observed in age, smoking history, mean systolic blood pressure, RDW_SD, RDW_CV, PLT, TG, TC, FT3, CRP, LAD, and LVEF between the AF and non-AF groups of Han patients (*P* < .05) (Table 1). Moreover, significant differences were noted in age, smoking history, drinking history, associated coronary heart disease, mean systolic blood pressure, RDW_SD, RDW_CV, PLT, TG, TC, HDL, LDL, FT3, CRP, and LAD between the AF and non-AF groups in the whole population (*P* < .05) (Table 2).

**Table 1:** Comparison of general data between AF and non-AF groups of Tibetan patients and Han patients.

	Tibetan patients			Han patients		
	Non-AF group	AF group	*P*	Non-AF group	AF group	*P*
Sex						
Man	34 (68%)	28 (56%)	.216	20 (40%)	23 (46%)	.545
Women	16 (32%)	22 (44%)		30 (60%)	27 (54%)	
Smoking history						
Yes	29 (58%)	37 (74%)	.091	31 (62%)	41 (82%)	.026
No	21 (42%)	13 (26%)		19 (38%)	9 (18%)	
Drinking history						
Yes	34 (68%)	44 (88%)	.016	38 (76%)	43 (86%)	.202
No	16 (32%)	6 (12%)		12 (24%)	7 (14%)	
Associated hypertension						
Yes	15 (30%)	14 (28%)	.826	11 (22%)	17 (34%)	.181
No	35 (70%)	36 (72%)		39 (78%)	33 (66%)	
Associated diabetes						
Yes	38 (76%)	41 (82%)	.461	37 (74%)	38 (76%)	.817
No	12 (24%)	9 (18%)		13 (26%)	12 (24%)	
Associated CHD						
Yes	30 (60%)	42 (84%)	.008	34 (68%)	42 (84%)	.061
No	20 (40%)	8 (16%)		16 (32%)	8 (16%)	
Age, y	55 (46–67.25)	69 (61–74)	<.001	55 (51.75–68)	75 (65.75–79)	<.001
WBC (×10^9^/L)	6.22 (5.06–7.75)	5.47 (4.05–7.22)	.075	5.46 (4.54–6.7)	5.58 (4.29–7.16)	.694
RBC (×10^12^/L)	5.02 ± 0.83	5.03 ± 0.81	.972	4.81 ± 0.67	4.62 ± 0.89	.226
Hb, g/L	152.64 ± 21.91	154.1 ± 21.24	.736	146.56 ± 23.36	144.76 ± 25.36	.713
RDW_SD, fL	44.9 (43.1–48.7)	49.4 (45.98–58.83)	<.001	45.05 (43.08–47.88)	48.25 (45.1–53.08)	<.001
RDW_CV (%)	13.4 (12.98–14.18)	14.65 (13.38–17.2)	<.001	13.2 (12.78–13.7)	13.7 (13.2–14.93)	.011
PLT (×10^9^/L)	192.5 (152.25–275.25)	146 (112.25–181.75)	<.001	178.5 (151–203.25)	151 (119–189.5)	.011
FBG, mmol/L	4.96 (4.62–5.95)	4.88 (4.54–5.8)	.51	4.94 (4.61–5.75)	5.33 (4.75–5.98)	.213
TG, mmol/L	1.25 (0.91–1.71)	0.95 (0.72–1.43)	.009	1.34 (1.14–2.28)	1.02 (0.79–1.34)	0
TC, mmol/L	4.14 (3.76–4.8)	3.41 (2.6–4.09)	<.001	3.88 ± 0.9	3.4 ± 1.11	.019
HDL, mmol/L	0.98 ± 0.19	0.84 ± 0.2	.001	1.04 (0.84–1.18)	0.92 (0.75–1.1)	.06
LDL, mmol/L	2.77 (2.18–3.36)	2.01 (1.43–2.61)	<.001	2.26 ± 0.79	2 ± 0.93	.126
UA, μmol/L	344 (280.75–417)	356 (318.5–427)	.328	328 (273–410.75)	340 (283.5–475.5)	.352
TSH, mIU/L	1.79 (1.15–4.17)	2.68 (1.27–4)	.637	2.8 (1.92–3.76)	2.57 (1.35–4.26)	.381
FT3, pmol/L	4.92 (4.29–5.3)	4.5 (3.51–4.99)	.001	5.07 ± 0.87	4.34 ± 0.66	<.001
FT4, pmol/L	11.43 (10.06–12.88)	12.4 (10.41–14.33)	.124	10.92 (9.85–12.38)	11.11 (10.34–13.53)	.19
CRP, mg/dL	0.23 (0.13–0.42)	0.61 (0.28–2.04)	<.001	0.11 (0.05–0.27)	0.36 (0.15–1.5)	<.001
LAD, mm	35.68 ± 5.04	45.98 ± 7.16	<.001	35 (31–38)	43 (39.5–50)	<.001
EDV, mL	110 (90.38–132.25)	115.5 (102–132.25)	.322	105.49 ± 23.23	110.23 ± 25.94	.338
IVST, mm	10 (9–11)	11 (10–12)	.009	11 (9.75–11)	10 (10–11)	.621
LVPWT, mm	9 (9–11)	11 (10–12)	.009	10 (10–11)	10 (9–11)	.221
LVEF (%)	63 (54–65)	62.5 (56–66)	.909	64 (61–68)	60.5 (55.75–65.25)	.01
Mean systolic pressure, mm Hg	125.3 ± 18.13	122.5 ± 17.27	.431	130 (119.5∼147.25)	120.5 (111.5∼130)	.002
Mean diastolic pressure, mm Hg	70 (66–82.5)	73.5 (67.75–83)	.411	77.56 ± 10.68	75.6 ± 8.66	.316

CRP = C-reactive protein, EDV = left ventricular end-diastolic dimension, FBG = fasting blood glucose, FT3 = free triiodothyronine, FT4 = free thyroxine, Hb = hemoglobin, HDL = high-density lipoprotein, IVST = interventricular septal thickness, LAD = left atrial diameter, LDL = low-density lipoprotein, LVEF = left ventricular ejection fraction, LVPWT = left ventricular posterior wall thickness, PLT = platelet, RBC = red blood cell count, RDW_CV = red cell distribution width, RDW_SD = red cell distribution width, TC = total cholesterol, TG = triglyceride, TSH = thyroid-stimulating hormone, UA = uric acid, WBC = white blood cell count.

**Table 2:** Comparison of general data between AF and non-AF groups in the whole population.

	Non-AF group	AF group	*Z*/*T*/*χ*^2^	*P*
Sex				
Man	54 (54%)	51 (51%)	0.180	.671
Women	46 (46%)	49 (49%)		
Smoking history				
Yes	60 (60%)	78 (78%)	7.574	.006
No	40 (40%)	22 (22%)		
Drinking history				
Yes	72 (72%)	87 (87%)	6.903	.009
No	28 (28%)	13 (13%)		
Associated hypertension				
Yes	26 (26%)	31 (31%)	0.613	.434
No	74 (74%)	69 (69%)		
Associated diabetes				
Yes	75 (75%)	79 (79%)	0.452	.502
No	25 (25%)	21 (21%)		
Associated CHD				
Yes	64 (64%)	84 (84%)	10.395	.001
No	36 (36%)	16 (16%)		
Age, y	55 (50–67.75)	72 (62–77)	–6.993	<.001
WBC (×10^9^/L)	5.72 (4.77–7.07)	5.51 (4.26–7.18)	–0.9	.368
RBC (×10^12^/L)	4.92 ± 0.76	4.83 ± 0.87	0.81	.419
Hb, g/L	149.6 ± 22.74	149.43 ± 23.74	0.052	.959
RDW_SD, fL	44.95 (43.1–48.18)	48.75 (45.73–55.53)	–5.79	<.001
RDW_CV (%)	13.3 (12.83–14.1)	14.25 (13.2–15.85)	–4.209	<.001
PLT (×10^9^/L)	184.5 (151.5–222.5)	147 (117.5–185.5)	–4.521	<.001
FBG, mmol/L	4.95 (4.63–5.89)	5.07 (4.68–5.94)	–0.446	.656
TG, mmol/L	1.3 (1.01–2.12)	1.01 (0.76–1.36)	–4.456	<.001
TC, mmol/L	4.02 (3.41–4.75)	3.31 (2.51–4.12)	–4.728	<.001
HDL, mmol/L	1 (0.83–1.15)	0.86 (0.73–1.02)	–3.561	<.001
LDL, mmol/L	2.58 (1.73–3.27)	2.01 (1.35–2.71)	–3.596	<.001
UA, μmol/L	338 (274.75–415.5)	351 (309–444.5)	–1.436	.151
TSH, mIU/L	2.55 (1.51–3.97)	2.65 (1.31–4.16)	–0.154	.878
FT3, pmol/L	4.96 ± 0.76	4.29 ± 0.81	5.976	<.001
FT4, pmol/L	11.13 (9.94–12.76)	11.79 (10.37–14.1)	–1.921	.055
CRP, mg/dL	0.18 (0.07–0.33)	0.45 (0.25–1.72)	–5.396	<.001
LAD, mm	36 (32–38)	44 (40–50)	–8.642	<.001
EDV, mL	107 (88.5–128)	114 (94.5–126)	–1.452	.147
IVST, mm	10 (9–11)	11 (10–12)	–1.677	.094
LVPWT, mm	10 (9–11)	10 (9–11)	–1.295	.195
LVEF (%)	63 (59–68)	62 (56–65.75)	–1.662	.097
Mean systolic pressure, mm Hg	128 (116–142)	120.5 (110.25–130)	–2.468	.014
Mean diastolic pressure, mm Hg	76 (69–83)	75 (70–81)	–0.11	.912

CRP = C-reactive protein, EDV = left ventricular end-diastolic dimension, FBG = fasting blood glucose, FT3 = free triiodothyronine, FT4 = free thyroxine, Hb = hemoglobin, HDL = high-density lipoprotein, IVST = interventricular septal thickness, LAD = left atrial diameter, LDL = low-density lipoprotein, LVEF = left ventricular ejection fraction, LVPWT = left ventricular posterior wall thickness, PLT = platelet, RBC = red blood cell count, RDW_CV = red cell distribution width, RDW_SD = red cell distribution width, TC = total cholesterol, TG = triglyceride, TSH = thyroid-stimulating hormone, UA = uric acid, WBC = white blood cell count.

**Figure 1:**
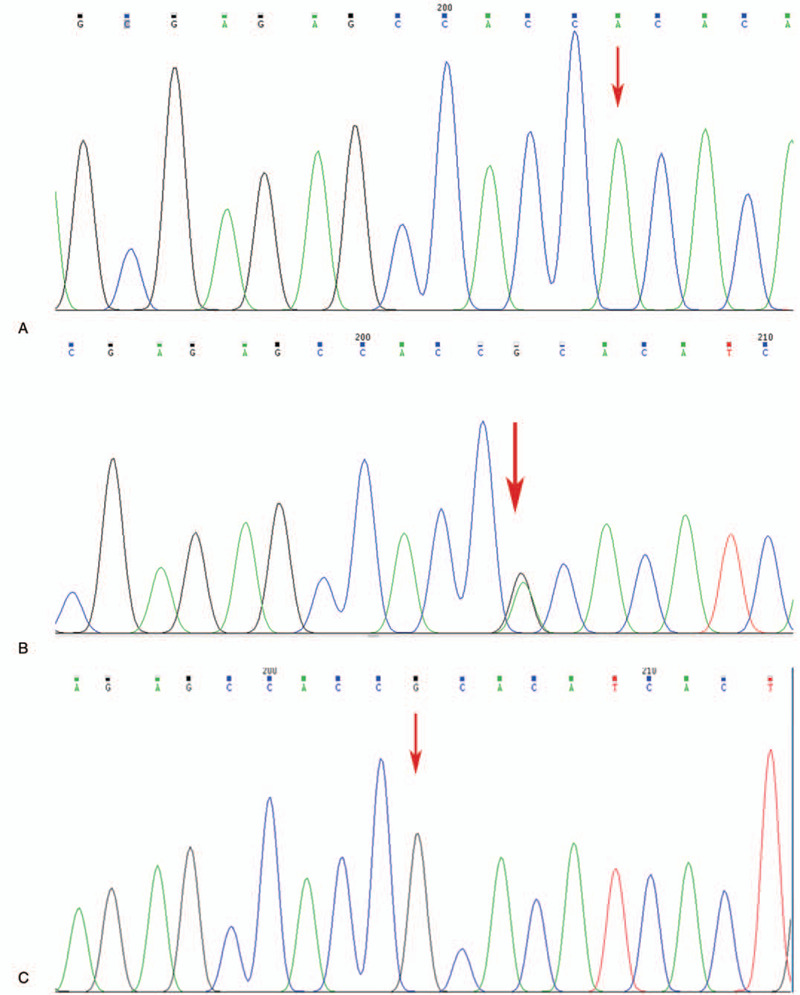
Sequencing map of gene mutation at the H558R locus of the *SCN5A* gene. A, AA (wild type); B, AG (heterozygous mutation); C, GG (homozygous mutation).

Three genotypes were detected in the H558R locus of the *SCN5A* gene of Tibetan and Han nationalities by gene sequencing: AA, AG, and GG (Fig. 1). After the Hardy–Weinberg equilibrium test, the genotype frequencies of the H558R locus of the *SCN5A* gene in the AF and non-AF groups of Tibetan and Han nationalities complied with the law of genetic balance and were constant (*P* > .05) (Table 3). The genotype and allele frequencies of the H558R locus of the *SCN5A* gene in the AF groups of Tibetan and Han nationalities and the whole population were significantly different from those in the non-AF groups (*P* < .05). The AG and GG genotype frequencies and G allele frequencies were higher in the AF groups than in the non-AF groups (Table 4). The genotype and allele frequency of the H558R locus of the *SCN5A* gene in the AF group of Tibetan nationalities were not significantly different from those in the AF group of Han nationalities (*P* > .05) (Table 5).

**Table 3:** Hardy–Weinberg equilibrium test in AF and non-AF groups.

	AF group				Non-AF group			
Genotype frequency	Observed value	Expected value	*χ* ^2^	*P*	Observed value	Expected value	*χ* ^2^	*P*
Tibetan			0.121	.941			2.429	.297
AA	9	8.405			36	34.445		
AG	23	24.19			11	14.11		
GG	18	17.405			3	1.445		
Han			0.006	.997			0.574	.751
AA	6	6.125			36	35.28		
AG	23	22.75			12	13.44		
GG	21	21.125			2	1.28		

AF = atrial fibrillation.

**Table 4:** Comparison in genotypes and allele frequencies between AF and non-AF groups in Tibetan, Han, and whole populations.

	Genotype of the H558R locus of the *SCN5A* gene			Allele	
Nationality	AA	AG	GG	A	G
Tibetan					
Non-AF group	36 (72%)	11 (22%)	3 (6%)	83 (83%)	17 (17%)
AF group	9 (18%)	23 (46%)	18 (36%)	41 (41%)	59 (59%)
*χ*^2^	31.150			37.436	
*P*	<.001			<.001	
Han					
Non-AF group	36 (72%)	12 (24%)	2 (4%)	84 (84%)	16 (16%)
AF group	6 (12%)	23 (46%)	21 (42%)	35 (35%)	65 (65%)
*χ*^2^	40.581			49.818	
*P*	<.001			<.001	
Whole population					
Non-AF group	72 (72%)	23 (23%)	5 (5%)	167 (83.5%)	33 (16.5%)
AF group	15 (15%)	46 (46%)	39 (39%)	76 (38%)	124 (62%)
*χ*^2^	71.284			86.823	
*P*	<.001			<.001	

AF = atrial fibrillation.

**Table 5:** Comparison in the H558R locus genotype of *SCN5A* gene and allele frequency between Tibetan and Han patients with AF.

	Genotype of the H558R locus of the *SCN5A* gene			Allele	
Nationality	AA	AG	GG	A	G
Tibetan patients with AF	9 (18%)	23 (46%)	18 (36%)	41 (41%)	59 (59%)
Han patients with AF	6 (12%)	23 (46%)	21 (42%)	35 (35%)	65 (65%)
*χ* ^2^	0.831			0.764	
*P*	.66			.382	

AF = atrial fibrillation.

The multivariate logistic regression analysis revealed that the associated coronary heart disease, LAD, IVST, LVPWT, and G alleles (odds ratio [OR] = 4.797, 95% confidence interval [CI] = 2.070–7.498, *P* = .014 < .05) were independent risks factors for the occurrence of AF in the Tibetan population (Table 6). Age, LAD, and G allele (OR = 5.844, 95% CI = 1.135–30.087, *P* = .035 < .05) were independent risk factors for AF in the Han population (Table 7). In the whole population, associated coronary heart disease (CHD) (OR = 10.897, 95% CI = 2.783–42.671, *P* = .001 < .05), age (OR = 1.080, 95% CI = 1.024–1.139, *P* = .004 < .05), TC (OR = 72.419, 95% CI = 1.499–3499.417, *P* = .030 < .05), LAD (OR = 1.240, 95% CI = 1.119–1.374, *P* < .05), and G allele (OR = 10.099, 95% CI = 3.304–30.869, *P* < .05) were independent risk factors for AF (Table 8).

**Table 6:** Logistic regression analysis of the Tibetan population.

	*B*	SE	Wald	*P*	OR	95% CI
Drinking history						
Yes	0.145	1.711	0.007	.933	1.156	0.040–33.040
No	0				1	
Associated CHD						
Yes	1.707	0.801	6.830	.009	5.568	1.244–9.952
No	0				1	
Age, y	0.058	0.046	1.632	.201	1.060	0.969–1.159
RDW_SD, fL	0.309	0.187	2.733	.098	1.362	0.944–1.965
RDW_CV (%)	–0.813	0.627	1.682	.195	0.443	0.130–1.515
PLT (×10^9^/L)	0.005	0.010	0.208	.648	1.005	0.985–1.025
TG, mmol/L	–2.347	1.653	2.015	.156	0.096	0.004–2.443
TC, mmol/L	5.656	4.109	1.895	.169	285.978	0.091–899255.891
HDL, mmol/L	–6.538	5.922	1.219	.270	0.001	0.000–158.864
LDL, mmol/L	–7.294	4.275	2.912	.088	0.001	0.000–2.957
FT3, pmol/L	–0.220	0.820	0.072	.788	0.802	0.161–4.007
CRP, mg/dL	0.273	0.346	0.622	0.430	1.314	0.667–2.589
LAD, mm	0.407	0.164	6.172	.013	1.502	1.090–2.070
IVST, mm	2.945	1.198	6.039	.014	19.004	1.815–198.984
LVPWT, mm	1.994	1.385	4.675	.031	7.344	2.513–12.756
H558R						
G	1.568	1.449	6.061	.014	4.797	2.070–7.498
A	0				1	
Constant	–23.453	13.947	2.828	.093	0.000	

CHD = coronary heart disease, CRP = C-reactive protein, FT3 = free triiodothyronine, HDL = high-density lipoprotein, IVST = interventricular septal thickness, LAD = left atrial diameter, LDL = low-density lipoprotein, LVPWT = left ventricular posterior wall thickness, PLT = platelet, RDW_CV = red cell distribution width, RDW_SD = red cell distribution width, TC = total cholesterol, TG = triglyceride.

**Table 7:** Logistic regression analysis of the Han population.

	B	SE	Wald	*P*	OR	95% CI
Smoking history						
Yes	0.782	0.996	0.618	.432	2.187	0.311–15.390
No	0				1	
Age, y	0.112	0.047	5.687	.017	1.119	1.020–1.226
RDW_SD, fL	0.167	0.137	1.491	.222	1.182	0.904–1.545
RDW_CV (%)	–0.300	0.401	0.561	.454	0.741	0.337–1.625
PLT (×10^9^/L)	–0.009	0.008	1.113	.291	0.991	0.976–1.007
TG, mmol/L	–0.029	0.443	0.004	.948	0.972	0.408–2.315
TC, mmol/L	0.881	0.601	2.152	.142	2.413	0.744–7.829
FT3, pmol/L	–0.965	0.562	2.953	.086	0.381	0.127–1.145
CRP, mg/dL	1.010	0.719	1.974	.160	2.747	0.671–11.244
LAD, mm	0.210	0.071	8.799	.003	1.234	1.074–1.418
LVEF (%)	0.107	0.062	2.985	.084	1.113	0.986–1.258
Mean systolic pressure, mm Hg	–0.029	0.032	0.848	.357	0.971	0.913–1.033
H558R						
G	1.765	0.836	4.459	.035	5.844	1.135–30.087
A	0				1	
Constant	–21.617	12.300	3.089	.079	0.000	

CRP = C-reactive protein, FT3 = free triiodothyronine, LAD = left atrial diameter, LVEF = left ventricular ejection fraction, PLT = platelet, RDW_CV = red cell distribution width, RDW_SD = red cell distribution width, TC = total cholesterol, TG = triglyceride.

**Table 8:** Logistic regression analysis of the whole population.

	*B*	SE.	Wald	*P*	OR	95% CI
Smoking history						
Yes	–0.239	0.616	0.151	.697	0.787	0.235–2.632
No	0				1	
Drinking history						
Yes	0.509	0.770	0.437	.509	1.664	0.368–7.532
No	0				1	
Associated CHD						
Yes	2.388	0.696	11.761	.001	10.897	2.783–42.671
No	0				1	
Age, y	0.077	0.027	8.075	.004	1.080	1.024–1.139
RDW_SD, fL	0.070	0.078	0.820	.365	1.073	0.921–1.249
RDW_CV (%)	–0.103	0.216	0.228	.633	0.902	0.591–1.377
PLT (×10^9^/L)	–0.004	0.005	0.537	.463	0.996	0.987–1.006
TG, mmol/L	–1.305	0.711	3.375	.066	0.271	0.067–1.091
TC, mmol/L	4.282	1.979	4.685	.030	72.419	1.499–3499.417
HDL, mmol/L	–5.339	2.729	3.827	.050	0.005	0.000–1.010
LDL, mmol/L	–4.106	1.949	4.437	.055	0.016	0.000–1.252
FT3, pmol/L	–0.470	0.367	1.640	.200	0.625	0.304–1.284
CRP, mg/dL	0.079	0.161	0.241	.623	1.083	0.789–1.486
LAD, mm	0.215	0.052	16.797	.000	1.240	1.119–1.374
Mean systolic pressure, mm Hg	–0.017	0.017	1.036	.309	0.983	0.951–1.016
H558R						
G	2.312	0.570	16.456	.000	10.099	3.304–30.869
A	0				1	
Constant	–13.551	5.356	6.400	.011	0.000	

CHD = coronary heart disease, CRP = C-reactive protein, FT3 = free triiodothyronine, HDL = high-density lipoprotein, LAD = left atrial diameter, LDL = low-density lipoprotein, PLT = platelet, RDW_CV = red cell distribution width, RDW_SD = red cell distribution width, TC = total cholesterol, TG = triglyceride.

**Figure 2:**
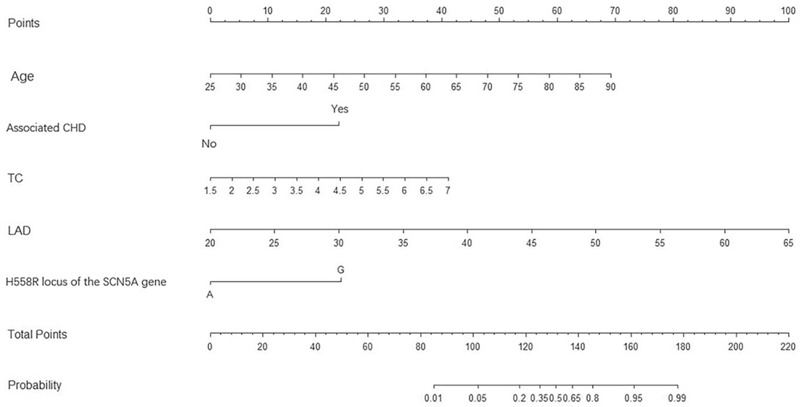
Alignment diagram for predicting the occurrence of AF at high altitude (2260 m). CHD = coronary heart disease, LAD = left atrial diameter, TC = total cholesterol.

Therefore, a nomogram (Han and Tibetan) was used to predict the occurrence of AF with independent risk factors, including associated CHD, age, TC, LAD, and G alleles (Fig. 2).

## 4 Discussion

Atrial fibrillation is one of the most common arrhythmias in cardiology. The complications caused by it will seriously damage the patient's quality of life (prone to cerebral infarction, heart failure, thromboembolism, etc), and increase the risk of cardiac death. According to epidemiological studies of AF, the prevalence of AF is still not paid attention to. The most important reason is the missed diagnosis and misdiagnosis of patients with paroxysmal and asymptomatic AF. As the population ages and other risk factors such as hypertension, diabetes, and cardiovascular diseases increase, it can be foreseen that the prevalence of AF will continue to rise in the future. Although anti-arrhythmic drugs, radiofrequency ablation and left atrial appendage closure have been widely used in clinical treatment, the pathogenesis of AF is still unclear at present, and due to frequent recurrences and serious complications of AF, the short-term and long-term effects of these drugs and surgery are not ideal. Therefore, the prevention of high-risk factors for AF is particularly important.

This study revealed 3 genotypes at the H558R locus of the *SCN5A* gene: AA, AG, and GG. The frequencies of AG and GG genotypes and G alleles were higher in the AF groups than in the non-AF groups, regardless of Tibetan or Han nationalities. However, no significant ethnicity-related difference was found between the Tibetan and Han patients with AF. The logistic regression analysis of the total population showed that associated CHD, age, TC, LAD, and G alleles were independent risk factors for AF occurrence. The G allele had 10.099 times higher risk of developing AF than the A allele (OR = 10.099, 95% CI = 3.304–30.869, *P* < .05).

Podzolkov et al^[9]^ proved that myocardial infarction is an important risk factor for the occurrence and development of AF in patients with CHD. In addition, the predictors of AF progression in patients with CHD include chronic heart failure, mitral insufficiency, and irreversible changes in regional myocardial left ventricular contraction. Similarly, Chamberlain et al^[10]^ reported that the risk of AF was significantly higher in patients with myocardial infarction than in the control group; it was an important predictor of AF. The results of this study were consistent with the findings of other related studies. In 1994, Benjamin et al^[11]^ used the Framingham cohort to identify independent risk factors for AF; age was the strongest predictor for the development of AF. The prevalence of AF increased from 0.5% in the 50 to 60 age group to 8.8% in patients aged >80 years.^[12]^ The result of this study also indicated that age was an independent risk factor for the occurrence and development of AF. This study also showed that the common risk factors for cardiovascular disease (e.g., diabetes, hypertension, and valvular disease) were associated with the increased risk of AF, and the changes in these risk factors reduced the incidence of AF.^[11]^ Significant differences were observed in TG, TC, HDL, and LDL between the AF and non-AF groups (*P* < .05). However, after logistic regression analysis of the whole population, only TC was shown to be an independent risk factor for AF. No unified conclusion exists regarding the relationship between lipids and AF. The results of each study were different; therefore, more related studies need to be performed in the future. The pathological mechanism of LAD enlargement is that the left ventricular end-diastolic pressure increases and the left ventricular compliance decreases, causing enhanced compensatory contraction of the left atrium, increasing the left atrial perfusion pressure, enlarging the left atrium, leading to electrical instability, and inducing AF occurrence. Relevant studies proved that AF started at LAD >31 mm, and LAD >45 mm led to permanent AF. Soeki et al^[13]^ also showed that LAD ≥45 mm was independently associated with the occurrence of AF. Similarly, Tang et al^[14]^ reported that LAD was significantly larger in the AF group than in the control group. Pearson correlation analysis of AF risk factors showed a positive correlation of the LAD level with AF, which was consistent with other findings. Thus, the results on general data in this study were comparable.

In 1995, George et al^[15]^ first localized the subunit encoded by the *SCN5A* gene to 3p21 by fluorescence in situ hybridization. The cardiac sodium channel is a glycosylated polypeptide complex composed of a core subunit and an auxiliary subunit. Sodium ion channels have the highest expression density on mammalian cardiomyocytes, with a distribution of >1 million. High-density-distributed sodium ion channels depolarize rapidly in the early stage of action potential (phase 0) and are responsible for the inward sodium current, which in turn causes the subsequent opening of each ion channel and plays a key role in the formation of an action potential. Abnormal expression or function of sodium channels on the cell membrane leads to disordered sodium ion influx, and the normal phase 0 depolarization cannot be formed. It affects the activity of ion channels in the late stage of action potential and results in the abnormal action potential and occurrence of arrhythmias. Studies have reported that *SCN5A* gene mutations cause a variety of arrhythmias, including long QT syndrome, Brugada syndrome, cardiac conduction system diseases, and AF.^[5]^ At present, 2 types of mutations are found in the *SCN5A* gene. One is functional acquired mutation and the other is loss-of-function mutation. Studies have shown that the occurrence of AF is associated with functional loss- or gain-of-function mutation of *SCN5A*.^[16–19]^*SCN5A* loss-of-function mutations further reduce sodium influx and shorten action potential duration, increasing the risk of AF.^[17]^ The gain-of-function mutation of *SCN5A* may promote AF by increasing the excitability of atrial myocytes. It promotes early postdepolarization by prolonging action potential duration or delays postdepolarization occurrence by promoting Na+ entry into atrial myocytes during the relaxation period.^[18,19]^

At present, relevant studies in China have shown that mutations in the A1673G locus of the *SCN5A* gene are associated with the occurrence of AF. The most common mutant genotype at the A1673G locus in patients with AF is GG, which is a high-risk factor for AF.^[20]^ Xie et al^[21]^ showed that the single-nucleotide polymorphism of the *SCN5A* gene in the Chinese Han population was significantly different from that in the American population. Similarly, Shadek et al^[22]^ found significant ethnicity-related differences in the H558R locus of the *SCN5A* gene between Han and Uygur patients with AF in Xinjiang. The G allele was found to be an independent risk factor for AF. The results of this study also showed that AG and GG genotypes were the most common genotypes of AF. The G allele was an independent risk factor for AF, which was consistent with other study. However, no significant ethnicity-related difference in AF was observed between Tibetan and Han nationalities. This might be related to the sample size, which needs further investigation in the future. Gene polymorphisms are not only related to AF, but also to other cardiovascular diseases. Related studies have shown that there is a significant association between the CYP11B2 (-344C/T) polymorphism and the -344T allele and essential hypertension. In addition, CYP11B2 -344C/T polymorphism and -344T alleles were also found to be associated with left ventricular hypertrophy.^[23,24]^

## 5 Conclusions

This study showed that combined CHD, age, TC, LAD, G alleles are independent risk factors for AF. There is a correlation between the occurrence of AF in Tibetan and Han nationalities at high altitudes and the H558R polymorphism of *SCN5A* gene; but there is no ethnic difference between the H558R polymorphism of *SCN5A* gene in Tibetan and Han nationalities. The results of this study provide a theoretical basis for future research on AF and *SCN5A* gene polymorphism in high-altitude Tibetan and Han nationalities at the molecular biology and genetic level.

This study had certain limitations. The sample size of the study was small. Therefore, more comprehensive studies with a large sample size should be conducted in the future. This study explored only the correlation between AF and polymorphism of the H558R locus of the *SCN5A* gene in Tibetan and Han nationalities at the same altitude, but did not investigate the influence of altitude on the polymorphism of H558R locus of the *SCN5A* gene. Hence, different altitude gradients should be used in future studies to explore their effect on the polymorphism of H558R locus of the *SCN5A* gene.

## Acknowledgment

In this study, Jiang Liu thank all the authors for providing the blood samples, clinical data, and data collection, collation, and analysis. At the same time, Jiang Liu also want to thank the Qinghai Provincial Department of Science and Technology and the Qinghai Provincial Health and Health Committee for their financial support for this institute.

## Author contributions

**Conceptualization:** Xiao Ling Su, Kaiyue Han.

**Data curation:** Kaiyue Han, Jinping Chai, Rong Wang, Sang Geng.

**Formal analysis:** Jinwei Zhang.

**Funding acquisition:** Xiao Ling Su.

**Investigation:** Yanmei Shen, Yuanfeng Ma.

**Methodology:** Yuanfeng Ma, Sang Geng.

**Project administration:** Dekuan Tian, Wei Li, Yanmei Shen.

**Resources:** Jinping Chai.

**Software:** Dekuan Tian, Jinwei Zhang, Rong Wang.

**Supervision:** Xiao Ling Su, Wei Li.

**Writing – original draft:** Jiang Liu, Fengcai Yao.

**Writing – review & editing:** Jiang Liu.
